# Description of two new species of *Rhaphidophora* and *Diestramima* (Orthoptera, Rhaphidophoridae) from China

**DOI:** 10.3897/zookeys.1275.181827

**Published:** 2026-03-31

**Authors:** Xue-Li Feng, Zheng-Xue Zhao, Jie Xiao, Xiao-Hui Song, Xiu-Dong Huang, Hao-Lin Chen

**Affiliations:** 1 College of Agriculture, Anshun University, Anshun, 561000, China College of Agriculture, Anshun University Anshun China; 2 Anshun Academy of Agricultural Sciences, Anshun, 562109, China Anshun Academy of Agricultural Sciences Anshun China

**Keywords:** Aemodogryllinae, Guizhou, new species, Rhaphidophorinae, taxonomy

## Abstract

The family Rhaphidophoridae (Orthoptera, Ensifera) was established by Walker in 1869 with *Rhaphidophora* Serville, 1838 as the type genus. Morphologically, all species are completely wingless, with a flattened, dorsally convex body, a longitudinally furrowed head vertex, slender legs, and foretibiae without tympana. Herein, two new species, *Rhaphidophora jiaozishanensis* Feng & Zhao, **sp. nov**. and Diestramima (Baculitettix) phytophylacis Feng & Zhao, **sp. nov**., are described and illustrated. Both new species were collected from Jiaozishan Town, Xixiu District, Anshun City, Guizhou Province, China. All specimens examined in this study are deposited in the collection of Anshun University, Anshun City, Guizhou Province, China.

## Introduction

The family Rhaphidophoridae is assigned to the suborder Ensifera of the order Orthoptera. In 1869, Walker established this family with *Rhaphidophora* Serville, 1838 as the type genus. All species within this family are entirely wingless, with a flattened body and a convex dorsal surface. The vertex of the head is divided by a longitudinal furrow, and the legs are relatively slender. Typically, the dorsal surface of the forecoxae bears small spines, whereas the foretibiae are devoid of tympana. The ovipositor is sickle-shaped, with the ventral margin of the ventral valves being either serrated or smooth. Regarding habitat preference, these species are gregarious and predominantly occupy dark, moist microhabitats such as in leaf litter, rock crevices, and caves (Rentz 1991).

Taxonomically, this family is divided into 10 subfamilies, including nine extant subfamilies and one fossil subfamily, encompassing 90 genera. Two subfamilies occur in China: Rhaphidophorinae and Aemodogryllinae. Collectively, these two subfamilies include 19 genera, of which eight belong to Rhaphidophorinae and 11 to Aemodogryllinae (Cigliano et al. 2025). These two subfamilies are readily distinguishable by key morphological traits: Rhaphidophorinae bears one movable long spine on the inner genicular lobe of the forefemur, with the longest dorso-apical spine surpassing that of the hind metatarsus; Aemodogryllinae, by contrast, has one movable long spine on the outer genicular lobe of the forefemur, and its longest dorso-apical spine does not exceed that of the hind metatarsus.

Serville (1838) established the genus *Rhaphidophora* Serville, 1838 with *R. picea* Serville, 1838, from Java Island, as the type species. This genus can be distinguished from other genera of Rhaphidophorinae by the presence or absence of a posterior projection on the male abdominal tergum and the degree of specialization of the epiproct. Currently, 116 species of *Rhaphidophora* have been reported from the Asian mainland, Pacific islands, and Australia, with only 27 species distributed in China (Liu and Zhang 2002; Bian et al. 2017; Qin et al. 2018; Liu et al. 2021; Lu et al. 2022; Di et al. 2024).

The genus *Diestramima* Storozhenko, 1990, with the type species *D. palpata* Rehn, 1906, was described in the subfamily Aemodogryllinae (Storozhenko 1990). Later, this genus was divided into three subgenera: *Baculitettix* Gorochov & Storozhenko, 2019; *Excisotettix* Gorochov & Storozhenko, 2019; and *Diestramima* s. str. (Gorochov and Storozhenko 2019). To date, 40 species of this genus have been recorded from China and Indochina, 34 of which are distributed in China (Storozhenko 1990; Gorochov and Storozhenko 1992, 2015, 2019; Gorochov 1994, 1998, 2002, 2010; Liu and Zhang 2001; Storozhenko and Dawwrueng 2014; Qin et al. 2016; Zhu and Shi 2018; Wang et al. 2019; Zhu et al. 2022, 2025; He et al. 2023).

In this study, two new species belonging to the genera *Rhaphidophora* and *Diestramima* are identified and described.

## Materials and methods

The new species were collected from Jiaozishan Town, Xixiu District, Anshun City, Guizhou Province, China (26.328947°N, 105.934672°E). The type specimens are deposited in the collection of Anshun University (**ASU**), Anshun City, Guizhou Province, China. Sampling was conducted in forested habitats by turning over fallen branches and leaf litter, followed by manual capture of the specimens.

Collected specimens were preserved in labelled centrifuge tubes containing 75% ethanol. Following identification, they were mounted on insect pins of sizes appropriate to their body dimensions. Pins were inserted slightly to the right or left of the pronotum midline. Due to the high risk of hind leg detachment during pinning, the specimens were handled with particular care. Once mounted, they were air-dried for 3–4 days at room temperature to prevent the growth of mould; however, this process results in a slight darkening of the body colouration.

The type specimens are deposited in the collection of Anshun University (ASU), Anshun City, Guizhou Province, China.

Morphological characteristics were examined under a Nikon SMZ25 stereomicroscope (Nikon Corporation, Tokyo, Japan). The acquired images were edited using Adobe Photoshop CC software (Adobe Systems Inc., San Jose, CA, USA).

The following conventions were employed for morphometric measurements: body – the distance from apex of vertical frons to posterior margin of the last abdominal tergite; pronotum – the distance from anterior margin to posterior margin of pronotum in the midline; fore femur – the distance from base to apex of the fore femur; hind femur – the distance from base to apex of the hind femur; ovipositor – the distance from base of subgenital plate to the apex of ovipositor. All measurements were taken to the nearest 0.01 mm using digital Vernier callipers (SHIKE Tools Co., Ltd, Hunan, China).

## Taxonomy

### Order Orthoptera Oliver, 1789

**Family Rhaphidophoridae Walker, 1869**

**Subfamily Rhaphidophorinae Walker, 1869**

**Tribe Rhaphidophorini Walker, 1869**

**Genus *Rhaphidophora* Serville, 1838**

#### Rhaphidophora jiaozishanensis

Taxon classificationAnimaliaOrthopteraRhaphidophoridae

Feng & Zhao sp. nov.

97DC9C6C-7D6A-5471-A7FB-755333D9387E

https://zoobank.org/8A8EC91D-3A81-4E64-8940-A50607386863

Figs 1, 2, 3

##### Chinese name.

轿子山驼螽.

##### Type material.

***Holotype***: China • 1 ♂; Guizhou Province, Anshun City, Xixiu District, Jiaozishan Town; 24 Aug. 2025; Xueli Feng & Zhengxue Zhao leg.; ASU ZooR20250802. ***Paratype***: China • 1 ♀; same data as holotype; ASU ZooR202508021.

##### Diagnosis.

*Rhaphidophora jiaozishanensis* sp. nov. can be distinguished from congeners by the morphological characteristics of its epiproct. While this new species shares certain similarities with *R. glenoides* Qin, Wang & He, 2024, the former has an obtusely rounded apex with a median depression extending from the base to the apex, but the latter has a relatively acute apex, with the median depression reaching only one-third of the length from the base. Additionally, the two species diverge in body colour and size, and the number of hind tibial spines. The new species exhibits a dark-brown body, possesses 20 inner and 21 outer spines on the hind tibia, and is larger in body size (♂ 24.7 mm, ♀ 19.24 mm). In contrast, *R. glenoides* displays a reddish-brown body, bears 17 or 18 spinules on each side of the hind tibia, and features a distinctly smaller body size (15 mm).

##### Description.

**Figure 1.**
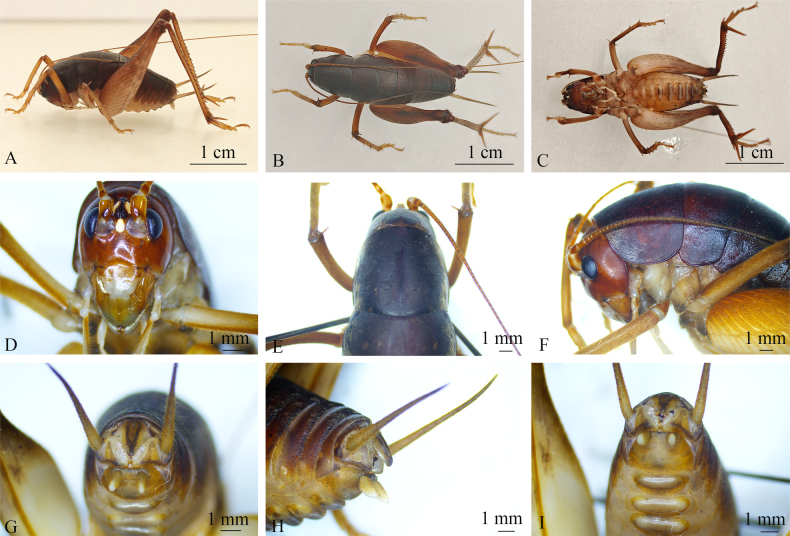
*Rhaphidophora jiaozishanensis* sp. nov. (male). **A**. Habitus, lateral view; **B**. Habitus, dorsal view; **C**. Habitus, ventral view; **D**. Head, frontal view; **E**. Head and pronotum, dorsal view; **F**. Head and thorax, lateral view; **G**. Terminalia, ventral view; **H**. Terminalia, lateral view; **I**. Terminalia, ventral view.

Male. Body rather large (24.7 mm, Fig. 1A–C). Fastigium of vertex divided by longitudinal furrow into pair of plate-like processes (Fig. 1D). Eyes reniform, near upper portion of outer margin of antennal sockets (Fig. 1D). Lateral ocelli oval, occupying approximately three-quarters of lateral margins of entire tubercles and situated at lateral base (Fig. 1D). Anterior margin of pronotum straight; posterior margin protruding caudad; ventral margin arched (Fig. 1E, F); posterior margin of mesonotum protruding caudad; posterior margin of metanotum protruding caudad (Fig. 1F).

Fore and mid legs slender; hind legs relatively long and robust. Dorsal surface of forecoxae bearing small spines. Fore femur about ^1^/_2_ times longer than pronotum, with ventral and dorsal sides unarmed; internal genicular lobe with 1 spine, and external genicular lobe without spine; fore tibia ventrally with 2 inner and 2 outer spines; apex with 1 pair of spines on ventral surface. Middle femur ventrally unarmed; internal and external genicular lobes with 1 long spine each on dorsal surface; dorsal surface of mesotibiae with 2 spines on both inner and outer sides, with 1 spine on each side of dorsal apex; ventral surface with 2 spines at middle and 1 spine on each side of ventral apex. Hind femur ventrally unarmed; internal genicular lobe with 1 spine; hind tibia dorsally with 20 inner and 21 outer spines, apex with 1 pair of long dorsal spines, 1 pair of long ventral spines, and 1 pair of small spines between paired ventral spines; the longest dorso-apical spine exceeds dorso-apical spine on hind metatarsus. Hind metatarsus keeled beneath.

Abdominal tergites without processes. Posterior margin of tenth abdominal tergite concave; abdominal sternites bearing oval ventral processes (Fig. 1C, I). Epiproct simple, approximately triangular, with a rounded apex and overall symmetry; base concave in an arcuate manner, with each lateral side of the base possessing an elliptical structure densely covered by fine setae. Epiproct with a longitudinal structure along midline, defined by raised margins on either side and a central depression, similarly densely adorned with setae (Fig. 1G, H).

**Figure 2.**
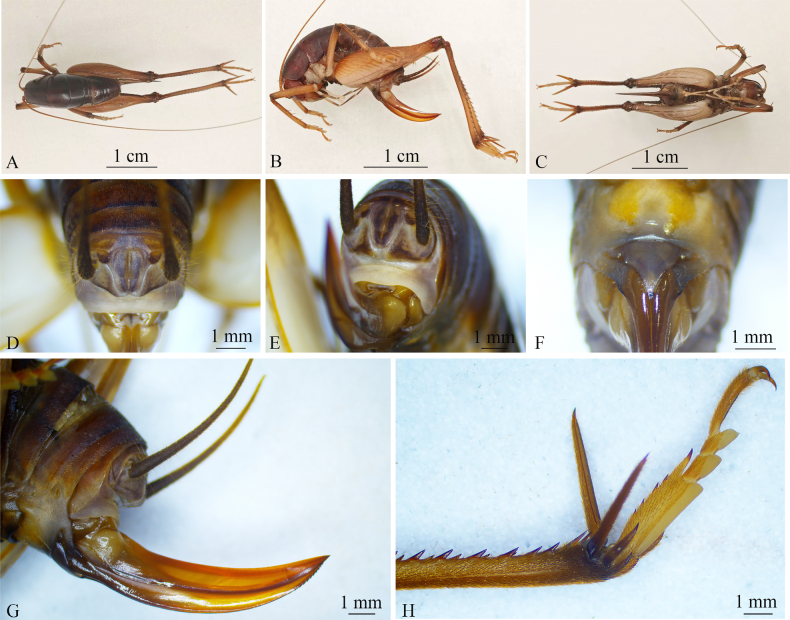
*Rhaphidophora jiaozishanensis* sp. nov. (female). **A**. Habitus, dorsal view; **B**. Habitus, lateral view; **C**. Habitus, ventral view; **D**. Apex of abdomen, posterodorsal view; **E**. Apex of abdomen, posterolateral view; **F**. Subgenital plate, ventral view; **G**. Ovipositor, lateral view; **H**. Hind tarsus, lateral view.

Female. General appearance similar to that of male (Fig. 2A–C), with distribution and quantity of leg spines consistent with male counterpart. Ovipositor longer than ½ length of hind femur; base of ovipositor broad, narrowing to apex; apex pointed and distinctly curved upwards; ventral edge of distal part of inferior valves bearing 12 distinct denticles (Fig. 2G). Subgenital plate furnished with 3 nearly triangular lobes; median lobe relatively large, with lateral margins parallel at basal fifth; paired lateral lobes smaller (Fig. 2F).

##### Colouration.

Body nearly unicolour, dark brown, and tergites shining. Face reddish brown, without distinct stripes. Legs with light-brown annular stripes.

##### Measurements.

Body: ♂ 24.7 mm, ♀ 19.24 mm; pronotum: ♂ 6.88 mm, ♀ 6.50 mm; fore femur: ♂ 8.10 mm, ♀ 7.72 mm; hind femur: ♂ 18.44 mm, ♀ 18.30 mm; hind tibia: ♂ 17.14 mm, ♀ 17.00 mm; ovipositor: ♀ 11.18 mm.

##### Distribution.

**Figure 3.**
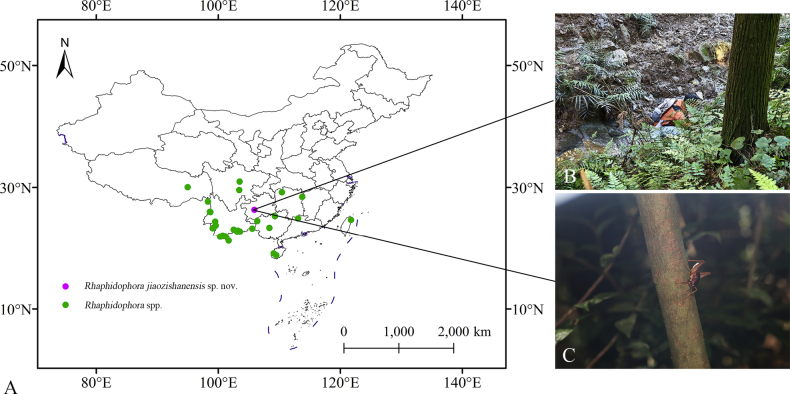
*Rhaphidophora jiaozishanensis* sp. nov. **A**. Distribution; **B**. Habitat; **C**. Female.

In China, most species of the genus *Rhaphidophora* are distributed south of the Yangtze River in the provinces of Hunan, Guangxi, and Yunnan. Among these, Yunnan is a distribution hotspot, with 11 recorded species (Fig. 3A). This new species was collected in Guizhou Province (Fig. 3A–C).

##### Etymology.

The specific epithet refers to the Jiaozishan Town where this species occurs.

### Subfamily Aemodogryllinae Jacobson, 1905

**Tribe Diestramimini Gorochov, 1998**

**Genus *Diestramima* Storozhenko, 1990**

**Subgenus *Baculitettix* Gorochov & Storozhenko, 2019**

#### Diestramima (Baculitettix) phytophylacis

Taxon classificationAnimaliaOrthopteraRhaphidophoridae

Feng & Zhao sp. nov.

B0048234-3F9B-5F45-B78C-0A2251EECDB4

https://zoobank.org/361EB7F4-3389-425B-A4D5-836F6EC4DC41

Figs 4, 5, 6

##### Chinese name.

植保突灶螽.

##### Type material.

***Holotype***: China • 1 ♂; Guizhou Province, Anshun City, Xixiu District, Jiaozishan Town; 24 Aug. 2025; Xueli Feng & Zhengxue Zhao leg.; ASU ZooD20250812. ***Paratypes***: China • 1 ♂; same data as holotype; ASU ZooD202508121. • 10 ♀; same data as holotype; ASU ZooD202508122 to ZooD202508129, ZooD2025081210, ZooD2025081211.

##### Diagnosis.

Diestramima (Baculitettix) phytophylacis sp. nov. can be distinguished from congeners based on the morphological features of the posteromedian process of the seventh abdominal tergite. Although this new species is similar to Diestramima (Baculitettix) beybienkoi Qin, Wang, Liu & Li, 2016, it is readily differentiated by the following diagnostic traits: in D. (B.) phytophylacis sp. nov., the posteromedian process of the seventh abdominal tergite is of moderate length, with nearly parallel lateral margins, and its apex is slightly broadened, forming an arcuate projection. In contrast, the posteromedian process of D. (B.) beybienkoi is relatively narrow, and its apex is distinctly dilated. Moreover, the posterior margin of the apex in D. (B.) beybienkoi is furnished with three small protuberances: the lateral protuberances are angular, whereas the median one is nearly semicircular.

##### Description.

**Figure 4.**
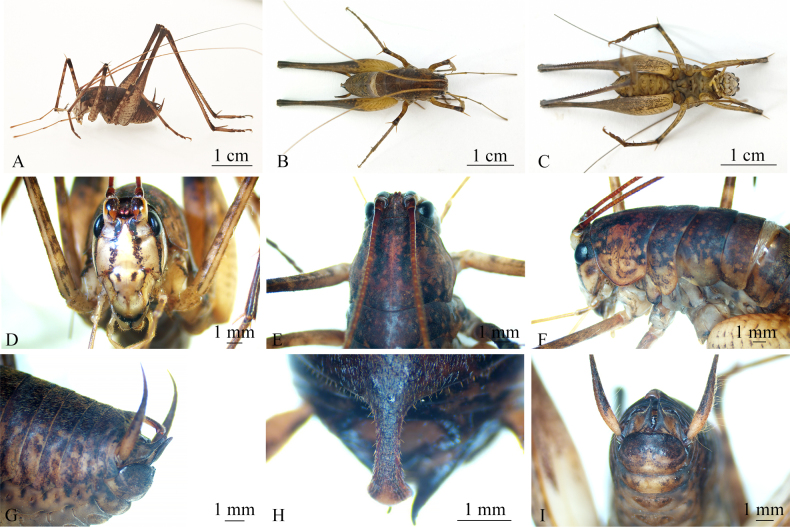
*Diestramima phytophylacis* sp. nov. (male). **A**. Habitus, lateral view; **B**. Habitus, dorsal view; **C**. Habitus, ventral view; **D**. Head, frontal view; **E**. Head and pronotum, dorsal view; **F**. Head and thorax, lateral view; **G**. Terminalia, lateral view; **H**. Posteromedian process of the seventh abdominal tergite, dorsal view; **I**. Terminalia, ventral view.

**Male**. Body medium-sized (22.16–23.14 mm, Fig. 4A–C). Fastigium of vertex divided by longitudinal furrow into a pair of plate-like processes (Fig. 4D). Eyes reniform, close to upper part of outer margin of antennal sockets (Fig. 4D). Lateral ocelli oval, occupying approximately 3/4 of lateral margins of entire tubercles, situated at lateral base; median ocellus located between antennal sockets (Fig. 4D). Anterior margin of pronotum extending forward, posterior margin protruding caudad, ventral margin arched (Fig. 4E, F); posterior margin of mesonotum protruding caudad; posterior margin of metanotum protruding caudad (Fig. 4F).

Fore and mid legs slender, hind legs relatively long and robust. Forecoxae bearing small, medial projections. Fore femur about 2.2 times longer than pronotum, with ventral and dorsal sides unarmed, external genicular lobe with 1 long spine, and internal genicular lobe without spine; fore tibia ventrally with 2 inner and 2 outer spines; apex with 1 dorsal spine and 1 pair of ventral spines, and 1 small spine between the paired ventral spines. Middle femur ventral and dorsal sides unarmed; internal and external genicular lobes with 1 long spine respectively on the dorsal surface; ventral surface of mesotibiae with 2 spines on both the inner and outer sides, apex with 1 pair of small dorsal spines and 1 pair of ventral spines, and 1 small spine between paired ventral spines. Hind femur with 7 or 8 inner spines on ventral surface, internal genicular lobe with 1 small spine; external genicular lobe unarmed. Hind tibia dorsally with 27–29 inner and 29–31 outer spines; subapex with 1 pair of dorsal spines, and apex with 1 pair of dorsal spines and 2 pairs of ventral spines, with intero-dorsal spine slightly shorter than hind basitarsus; longest dorso-apical spine not exceeding dorso-apical spine of hind metatarsus. Hind metatarsus keeled beneath.

Posterior margin of sixth abdominal tergite nearly straight. Posteromedian process of seventh abdominal tergite of medium length, with lateral margins nearly parallel; apex slightly broadened and forming an arcuate projection (Fig. 4G, H). Basal half of paraproct expanded, tapering posteriorly, and with a blunt apex (Fig. 4G).

**Figure 5.**
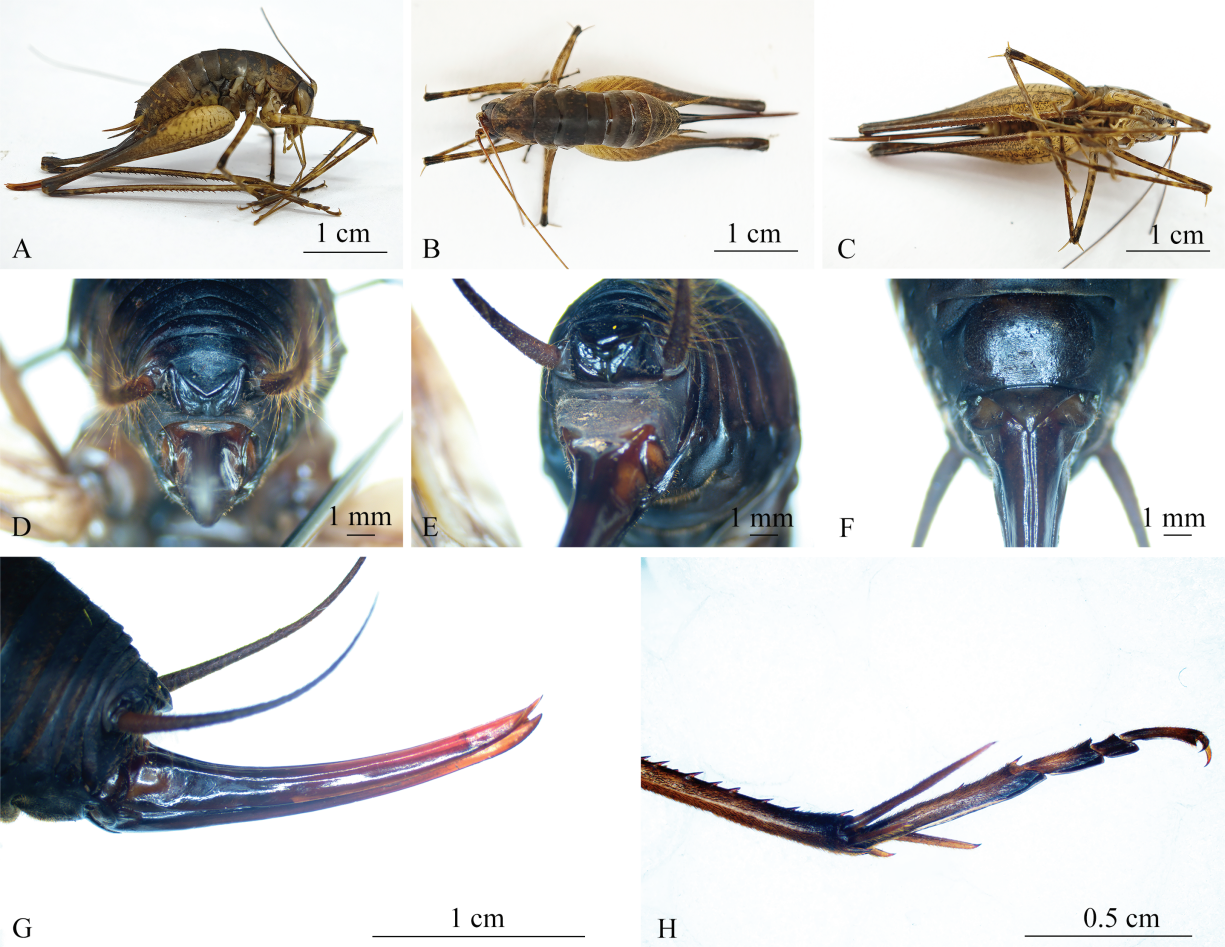
*Diestramima phytophylacis* sp. nov. (female). **A**. Habitus, lateral view; **B**. Habitus, dorsal view; **C**. Habitus, ventral view; **D**. Apex of abdomen, posterodorsal view; **E**. Apex of abdomen, posterolateral view; **F**. Subgenital plate, ventral view; **G**. Ovipositor, lateral view; **H**. Hind tarsus, lateral view.

**Female**. General appearance similar to males (Fig. 5A–C). Ovipositor length nearly equal to body length, with a broad base, narrowing to apex, pointed and slightly curved upwards, almost indistinct denticles on ventral edge of distal part of inferior valves (Fig. 5G). Subgenital plate roughly triangular (Fig. 5F).

##### Colouration.

Body blackish brown, mottled with dark-brown blotches. Head light brown, with a pair of dark vertical bands beneath eyes and at antennal sockets, dark dorsal spots posterior to eyes, and aclypeus bearing one dark spot on each side. Legs with light-brown annular stripes.

##### Measurements.

Body: ♂ 22.16–23.14 mm, ♀ 19.22–22.08 mm; pronotum: ♂ 6.00–6.46 mm, ♀ 5.80–6.18 mm; fore femur: ♂ 12.5–14.30 mm, ♀ 12.00–14.12 mm; hind femur: ♂ 24.04–25.80 mm, ♀ 23.54–25.00 mm; ovipositor: ♀ 16.48–20.90 mm.

##### Distribution.

**Figure 6.**
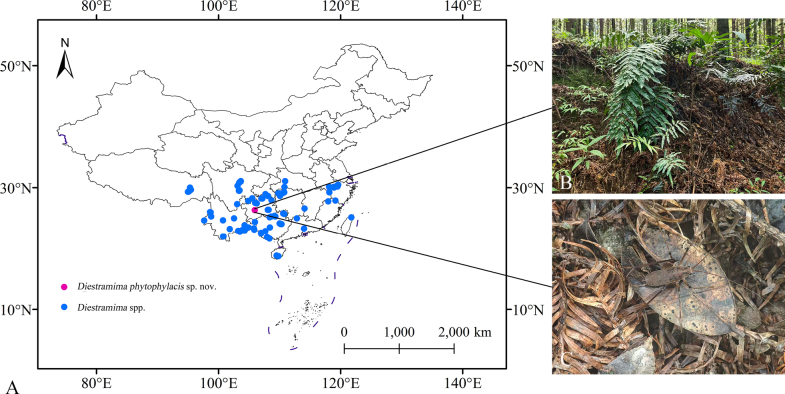
*Diestramima phytophylacis* sp. nov. **A**. Distribution; **B**. Habitat; **C**. Male.

Most species of this genus are distributed in southern China, including Hunan, Guangxi, Zhejiang, Yunnan, and Guizhou. Guangxi is a hotspot for *Diestramima* species, with 15 recorded (Fig. 6A). This new species was collected from Guizhou Province (Fig. 6A–C).

##### Etymology.

The specific epithet *phytophylacis* is a noun in the genitive singular, derived from the Greek roots *phyton* (meaning “plant”) and *phylax* (meaning “protector”). The name, meaning “of the plant protector,” is dedicated to the Plant Protection majors at the College of Agriculture, Anshun University, amd commemorates the discovery of the species during their field internship.

## Supplementary Material

XML Treatment for Rhaphidophora jiaozishanensis

XML Treatment for Diestramima (Baculitettix) phytophylacis

## Additional information

### Use of AI

This manuscript only uses artificial intelligence tools for linguistic proofreading and sentence refinement, and does not use artificial intelligence for image generation or the writing of the core content of the paper.

### Author ORCIDs

Xue-Li Feng https://orcid.org/0000-0002-3571-5669

Zheng-Xue Zhao https://orcid.org/0000-0003-1764-8690

Jie Xiao https://orcid.org/0009-0003-2004-2259

Xiao-Hui Song https://orcid.org/0009-0007-1782-9009

Xiu-Dong Huang https://orcid.org/0000-0002-1522-4811

Hao-Lin Chen https://orcid.org/0009-0002-9165-9347

### Data availability

All of the data that support the findings of this study are available in the main text.
